# Obstructive hydrocephalus caused by colloid cyst and treatment –resistant schizophrenia

**DOI:** 10.1192/j.eurpsy.2025.1193

**Published:** 2025-08-26

**Authors:** S. Laabidi, O. Laabidi, A. Touiti, A. Aissa, N. Bram

**Affiliations:** 1 forensic psychiatry; 2 psychiatry A, Razi hospital, manouba, Tunisia

## Abstract

**Introduction:**

Treatment-resistant schizophrenia can be of primay or secondary etiology. Systematic and thorough differential diagnostics is essential to exclude organic causes for treatment-resistant schizophrenia .Colloid cysts are congenital benign tumor accounting for 15-20 % of intraventricular mass but only about 1% of intracranial ones.They frequently cause psychiatric disturbances. The pathology behind these psychiatric symptoms remains unclear.

**Objectives:**

Through a case report and a review of the literature, we hypothesize that a colloid cyst in the third brain ventricle is the cause of resistance in a patient with treatment-resistant schizophrenia.

**Methods:**

Starting from a case report, we conducted a literature review on “PubMed”, using key words “colloid cyst and psychosis”, “colloid cyst and treatment-resistant schizophrenia”,

**Results:**

We present a 48-year-old male who has a family history of malignant neoplasm.There was no history of physical illness. His past psychiatric history revealed a diagnosis of schizophrenia, having been admitted several times in different inpatient psychiatric wards.In the psychiatric examination, the presence of auditory hallucinations, dissociated thinking, and predominantly negative symptoms was observed.Recently, he has been diagnosed with treatment-resistant schizophrenia owing to the inadequate response to two sequential antipsychotic trials (with adequate dose, duration, and adherence). After a 2-month hospitalization, the severity of the psychotic symptoms had decreased but did not show remission. With no prodromes or triggering factors, our patient presented a drop attack without loss of consciousness and with instantaneous recovery to baseline status. He did not have any of the same experience previously.The physical and neurological examination did not reveal any positive findings. All biochemistry parameters were reported as normal range. Following the evaluation process, an urgent head CT scan showed a colloidal cyst at the anterior end of the third ventricle with dilatation of the lateral ventricles. A cerebral MRI was performed in order to get a more detailed image; it confirmed the diagnosis of a third ventricle colloid cyst immediately adjacent to the foramen of Monro with obstructive hydrocephalus. The patient was referred to the neurosurgical department for further evaluation. This neurological tumor didn’t require neurosurgery.

**Conclusions:**

Our case implies the importance of neuroimaging in patients with treatment-resistant schizophrenia to rule out any underlying organic cause.It also emphasises the importance of considering an organic cause like any space occupying lesion in the brain (colloid cyst in the third brain ventricle in our case) for induction of psychopathological symptoms, even those of treatment-resistant schizophrenia.

**Disclosure of Interest:**

None Declared

